# The burden of medically indicated preterm birth due to preeclampsia and its association to the fullpiers score in a high-risk maternity setting

**DOI:** 10.61622/rbgo/2026rbgo50

**Published:** 2026-09-23

**Authors:** Giorgio Conte Tondello, Júlia Smânia Carvalho Ramos, Eloisa Celestino Shigeoka Dacaz, Beatryz Eyng dos Santos, Ana Clara Guimarães Justo Pereira, José Paulo de Siqueira Guida, Maria Laura Costa

**Affiliations:** 1 Universidade da Região de Joinville Joinville SC Brasil Universidade da Região de Joinville, Joinville, SC, Brasil.; 2 Maternidade Darcy Vargas Joinville SC Brasil Maternidade Darcy Vargas, Joinville, SC, Brasil.; 3 Universidade Estadual de Campinas Departamento de Ginecologia e Obstetrícia Campinas SP Brasil Departamento de Ginecologia e Obstetrícia, Universidade Estadual de Campinas, Campinas, SP, Brasil.

**Keywords:** Preeclampsia, Proteinuria, Pregnancy hypertension, Maternal-fetal exchange, Pregnancy induced, Infant, premature, Infant, newborn, Eclampsia, Maternal death, Fetal death

## Abstract

**Objective:**

Describe the prevalence of medically indicated preterm birth due to preeclampsia (PE), maternal and perinatal outcomes, and the use of the fullPIERS calculator for risk assessment.

**Methods:**

This retrospective cross-sectional study was conducted at a high-risk maternity center through a review of women records with medically indicated preterm birth due to PE between January 2020 and December 2023. Sociodemographic data, maternal and perinatal outcomes were evaluated. The fullPIERS calculator was applied using a cutoff value ≥2.15%, proposed as a predictor of adverse outcomes.

**Results:**

During the study period, 24,507 births were recorded, which 2,523 were preterm (10.2%). Preterm birth attributed to PE occurred in 9.75% of cases (n=246), with 2.4% (n=6) progressing to eclampsia. The mean maternal age was 31.7 years and mean systolic/diastolic blood pressures were 157.3 mmHg and 100.9 mmHg, respectively. Thrombocytopenia was observed in 25.6% of women, and elevated transaminases occurred in 14%. The mean gestational age at delivery was 34.06 weeks, and 68.8% of deliveries were indicated due to worsening blood pressure control. The stillbirth rate was 2.45% (n=6). Women with fullPIERS scores ≥2.15% (n=53) presented greater disease severity, including higher rates of thrombocytopenia, elevated creatinine (15%), lower gestational age at delivery (mean 32.8±3.2 weeks), and increased prevalence of HELLP syndrome (9.3%) and perinatal mortality (2.45%).

**Conclusion:**

Preeclampsia is an important cause of medically indicated preterm birth and is associated with adverse maternal and perinatal outcomes. The fullPIERS model may represent a useful adjunct tool for assessing disease severity and supporting clinical decision-making in this context.

## Introduction

Preeclampsia (PE) is a complex multisystem disorder defined by elevated blood pressure and multiorgan dysfunction, including the liver, kidneys, brain, or placenta.^(1-4)^ PE is one of the leading causes of maternal and perinatal morbidity and mortality, affecting up to 16% of pregnancies worldwide and resulting in approximately 500,000 preterm births annually.^(3)^ The complications of PE include severe conditions such as HELLP syndrome (hemolysis, elevated liver enzymes, and low platelet count), placental abruption, stroke, hepatic hematoma, acute pulmonary edema, myocardial infarction, renal failure, and eclampsia. These complications signiﬁcantly contribute to maternal and perinatal morbidity and mortality. A key component of PE treatment is defining the accurate moment for childbirth, with delivery of the feto-placental unit. The timing of delivery represents one of the greatest challenges in obstetric practice, especially in cases of early-onset PE.

In this context, the fullPIERS calculator, an available decision-support tool, was designed to identify pregnant women at increased risk of adverse maternal outcomes and to predict complications occurring within 48 hours to 7 days after assessment.^(5-10)^ It considers factors such as gestational age, chest pain, dyspnea, oxygen saturation, platelet count, creatinine, and liver enzymes.^(9,11)^ Despite its wide applicability in obstetric practice, there is heterogeneity in medical literature regarding the best cutoff value.

The present study aimed to identify cases of with medically indicated preterm birth due to PE, describe maternal and perinatal outcomes and apply the fullPIERS model, and to assess worse maternal and neonatal outcomes according to the cutoff evaluated in other studies in the Brazilian population.

## Methods

This is a retrospective, observational, descriptive, cross-sectional study conducted at Maternidade Darcy Vargas – Joinville, SC, Brazil. The facility is located in Joinville, Santa Catarina, a reference center for high-risk obstetrics and neonatology in the northern and northeastern regions of the state, accounting for approximately 500 births monthly, including both low-risk and high-risk pregnancies. The unit has specialized high-risk pregnancy clinics focusing on arterial hypertension, as well as clinics dedicated to postpartum PE follow-up and an inpatient sector for pregnant women with PE. According to the institution's protocol, all patients diagnosed with preeclampsia are managed as inpatients until clinical resolution.

Participants were selected among women who had preterm deliveries at the considered institution during the study period. Inclusion criteria were women with gestational age between 20 weeks and 36+6 weeks, in which delivery was indicated due to complications associated to hypertensive disease, including: hypertensive crisis, clinical signs of severe disease (Severe headache, visual changes (scotomas, blurred vision, diplopia, photopsia), intense pain in the right hypochondrium or epigastric pain, reduced level of consciousness, signs of respiratory dysfunction, and chest pain.), laboratory abnormalities, HELLP syndrome, acute pulmonary edema, or eclampsia and fetal indication (including non-reassuring cardiotocography, abnormal Doppler ﬁndings, or suspected placental abruption.

For the sample size calculation, an average of 500 monthly deliveries at Maternidade Darcy Vargas was considered, totaling 24,000 deliveries during the data collection period. Additionally, an average prevalence of PE in Brazil between 1.5-7% was assumed, based on previously published data.^(5,6)^ The prevalence of medically indicated births due to PE is not clear in the literature and therefore could not support calculation. To achieve a 97% signiﬁcance level, a conﬁdence interval calculation for a proportion indicated that 267 medical records were required to ensure statistically representative results. The sample size calculation was performed using the online tool available at http://estatistica.bauru.usp.br.

Data was collected using a form created in Excel spreadsheet. Only researchers directly involved in the study had access to the spreadsheet, for both data entry and analysis. All medical records meeting the inclusion criteria were analyzed and logged in the spreadsheet. Researchers used electronic devices with internet access to enter data into the spreadsheet.

The following variables were assessed: maternal age (in years at delivery); parity (nulliparous or multiparous); gestational age at delivery (>20 weeks and <37 weeks) obtained from medical records; diagnosis of preeclampsia as recorded by the healthcare team; blood pressure on admission (systolic and diastolic); length of hospital stay; laboratory tests (platelets <100,000, AST/ALT >70, creatinine ≥1.0 mg/dL); oxygen saturation at admission, indication for preterm delivery (HELLP syndrome, refractory hypertension, laboratory abnormalities, warning signs, or eclampsia); fullPIERS score was calculated, using latest data in the medical record before childbirth, to correlate with the indication, and applying on the online calculator (https://pre-empt.obgyn.ubc.ca/home-page/past-projects/fullPIERS/); presence of eclampsia, deﬁned as a generalized tonic-clonic seizure; birth weight; APGAR scores; fetal and maternal death.

Data were analyzed using EpiInfo 7 software (Centers for Disease Control – CDC, Atlanta, GA, USA). Descriptive analysis was performed using frequency and percentage for categorical variables and mean and standard deviation for numerical variables. Categorical variables were compared between groups with fullPIERS <2.15% and fullPIERS ≥2.15% (cut-off proposed as a reliable predictor of adverse perinatal outcomes) using Pearson's chi-square test, while numerical variables were analyzed using the Student's t-test.16 Length of hospital stay among different degrees of prematurity was evaluated using the Kruskal-Wallis test. All statistical tests were performed with a signiﬁcance level of α = 0.05, corresponding to a 95% conﬁdence interval.

To investigate if the FullPIERS model could be of use in predicting eclampsia, and if there was a cutoff point with increased association to such outcome, we further tested the model's discriminative performance using receiver operating characteristic (ROC) curve analysis. The area under the curve (AUC) was calculated to quantify overall discrimination, with the diagonal line representing chance performance. No optimal cutoff point was predefined, as the analysis was intended solely to assess model discrimination.

This study was submitted to and approved by the local Research Ethics Committee number: #7.213.015 (protocol # 83675424.1.0000.5363).

## Results

A total of 24,507 births were evaluated in the selected period, of these, 2.523 records of women with preterm birth. The prevalence of PE was 9.75% (n=246), of these women, the fullPIERS calculation was retrospectively evaluated, using latest data in the medical record before the delivery, to correlate with the indication for pregnancy termination. It was evidenced that 193 (78.5%) women presented a calculation of fullPIERS <2.15% and 53 (21.5%) women ≥2.15%. The mean fullPIERS score at admission was 2.30 The study population is shown in figure 1.

**Figure 1:**
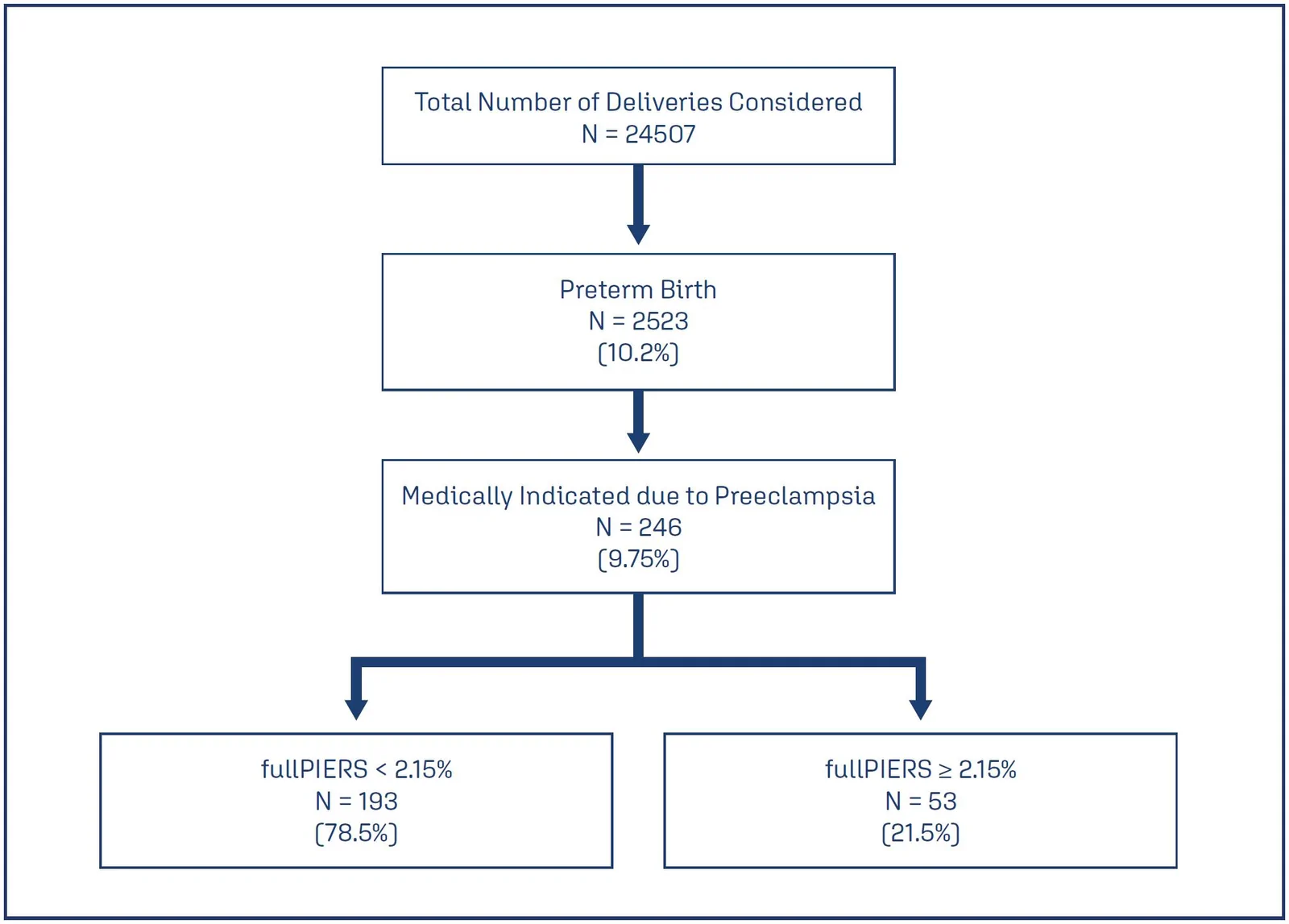
Study Population and included women for analysis

Regarding maternal-fetal outcomes, described in (Table 1), the prevalence of eclampsia was 2.4% (n = 6). The mean maternal age was 31.77 years (±7.03). Regarding parity, 52.8% were multiparous. The mean systolic and diastolic blood pressures were 157.3 mmHg and 100.9 mmHg, respectively. The mean interval from admission until delivery was 7.7 days (SD 6.1). Women presenting platelet count <150,000/μL were 25.61%. Aspartate Aminotransferase (AST) exceeding 70 U/L were 14.91% (35) and ALT (Alanine Aminotransferase) among 14.23% of cases (24). The creatinine level over 1 mg/dL was evidenced in 16.26% of the considered cases (40 women).

**Table 1:** Sociodemographic characteristics, Clinical, laboratory, and perinatal outcome of women with preeclampsia and medically indicator preterm birth

Variables	Mean ± SD; n%
	n=246
Age (years)	31.77±7,03
Parity	
Nulliparous	116(47.2)
Multiparous	130(52.8)
Systolic blood pressure (mmHg)	157.3±18.8
Diastolic blood pressure (mmHg)	100.9±15.8
Length of stay until delivery (days)	7.7±6.1
Platelets (/μL)	194413±71361
Platelets <150.000	63(25.61)
AST (U/L)	63.6±17.,3
AST >70	35(14.23)
ALT (U/L)	53.1±107.6
ALT >70	24(14.91)
Creatinine (mg/dL)	0.75±0.40
Creatinine >1	40(16.26)
Oxygen saturation (%)	98.02±0.95
Hydralazine dose (mg/kg)	4.18±3.68
Nifedipine dose (mg/kg)	1.31±2.23
Magnesium sulfate use	215(87.0)
Corticosteroid use	88(35.8)
Gestational age at delivery (weeks)	34.06±2.43
Causes of preterm birth*	
Hypertensive crisis	170(68.8)
Warning sign	27(11.0)
Laboratory alteration	27(11.0)
HELLP syndrome	23(9.3)
Eclampsia	6(2.4)
Acute pulmonary edema	1(0.4)
Birth weight (g)	2171.1±659.7
Birth weight <1,500 g	38(15.70)
Apgar 1st minute ≤7	111(45.87)
Apgar 5 th minute ≤7	33(13.64)
Fetal death**	6(2.45)
FullPIERS	2.30±4.97
FullPIERS >2.15%	53(21.54)

SD - standard deviation; AST - aspartate aminotransferase; ALT - alanine aminotransferase.

* Patients could present more than one cause of preterm birth.

** Fetal and neonatal death

The mean gestational age at delivery was 34.06 weeks, with 68.8% of preterm deliveries due to elevated blood pressure. The mean birthweight was 2,171.1 g, with 15.7% of newborns weighing <1,500g. Five-minute Apgar scores ≤7 occurred in 13.64%. The perinatal death rate was 2.45% (Table 1).

Comparative analysis between the fullPIERS <2.15% group (78.46%; n=193) and the fullPIERS ≥2.15% group (21.54%; n=53) is presented in table 2. In the fullPIERS ≥2.15% group, there was a signiﬁcant difference in the frequency of platelet count (with higher thrombocytopenia (p<0.001). There were also elevations in AST levels (p<0.001) and creatinine (p<0.001), as well as a higher frequency of AST >70 (p<0.001) and creatinine >1 (p=0.031). In addition, oxygen saturation was lower in the fullPIERS ≥2.15% group (p<0.001).

**Table 2:** Comparison of clinical characteristics between groups with fullPIERS <2.15% and ≥ 2.15%

Variables		fullPIERS <2.15%	fullPIERS >2.15%	p-value
N (%)		193(78.46)	53(21.54)	
Age (years)		31.67±7.02	32.11±7.12	0.691
Systolic Blood Pressure (mmHg)		156.8±18.3	158.7±20.3	0.544
Diastolic Blood Pressure (mmHg)		100.3±16.0	102.7±15.0	0.311
Hospital stay until delivery (days)		7.7±5.7	7.3±7.2	0.704
Platelets (units)		214008±64496	123056±44939	<0.001
Thrombocytopenia		21(10.88)	42(79.25)	<0.001
AST (U/L)		57,6±184.8	85.5±127.8	<0.001
	AST >70	18(9.33)	17(32.08)	<0.001
ALT (U/L)		46.9±112.0	73.6±89.7	0.140
	ALT >70	12(9.68)	12(32.43)	0.002
Creatinine (mg/dL)		0.59±0.27	0.76±0.82	<0.001
	Creatinine > 1	10(5.18)	8(15.09)	0.031
Oxygen saturation (%)		98.2±0.7	97.3±1.3	<0.001
Hydralazine dose (mg/kg) †		4.28±3.78	3.85±3.27	0.599
Nifedipine dose (mg/kg)		1.36±2.35	1.09±1.49	0.662
Magnesium sulfate use §		163(84.9)	52(96.3)	0.026
Corticosteroid use‡		62(32.3)	26(48.1)	0.032
Eclampsia		5(2.59%)	3(5.66)	0.374
Gestational age at delivery (weeks)		34.4±2.06	32.8±3.2	0.001
Causes of preterm delivery*				
	Hypertensive crisis	145(75.5)	25(46.3)	<0.001
	Warning signs	33(17.2)	7(13,0)	0.457
	Laboratory alteration	15(7.8)	12(22.2)	<0.001
	HELLP	9(4.7)	14(25.9)	<0.001
	Eclampsia	3(1.6)	3(5.6)	0.093
	Acute pulmonary edema	0(0.0)	1(1.9)	0.059
Birth weight (g)		2241±620	1319±736	0.004
	Birth weight <1500g	22(11,58)	16(30.77)	0.002
Apgar 1st minute ≤7		91(47.64)	20(39.22)	0.360
Apgar 5th minute ≤7		27(14.14)	6(11.76)	0.834
Fetal death		2(1.04)	4(7.69)	0.024

Data are presented as mean ± standard deviation or frequency and percentage. Bold values indicate statistical significance (p<0.05). AST - aspartate aminotransferase; ALT - alanine aminotransferase.

* Patients could present with more than one cause of preterm delivery.

† Number of doses of the medications used before the termination of pregnancy

§ Due to PE with severe features

‡ For fetal lung maturation

Regarding obstetric outcomes, gestational age at delivery was signiﬁcantly lower in the fullPIERS ≥2.15% group (p=0.001). Among the causes of preterm delivery, laboratory abnormalities and HELLP syndrome were signiﬁcantly associated with fullPIERS ≥2.15% (both p=0.001). Conversely, hypertensive crisis was more frequent in the fullPIERS <2.15% group (75.5%). The prevalence of birth weight <1500 g was also higher in the ≥2.15% group (p=0.002). Furthermore, stillbirth was associated with the ≥2.15% group (p=0.024).

Table 3 presents the analysis of the length of hospital stay from admission until delivery across different degrees of prematurity. According to the Kruskal-Wallis test, the length of stay showed a statistically signiﬁcant difference among groups with different gestational ages (p = 0.001). Women with moderate prematurity had the longest mean number of hospitalization days (11.46 days) compared to those classiﬁed as very preterm (6 days) and late preterm (7.13 days).

**Table 3:** Length of hospital stay from admission until delivery

Variables	n	Mean ± SD	p-value
		Length of hospital stay until delivery	
Late preterm	177	7.13±5.54	0.081a,b
Moderate preterm	55	9.71±7.46	
Extreme preterm	12	6.33±4.67	

Late preterm: 34–36+6 weeks; moderate preterm: 32–33+6 weeks; very preterm: 28–31+6 weeks; extreme preterm: <28 weeks. Values obtained using the Kruskal-Wallis test.

a,b Different letters represent statistically signiﬁcant differences after pairwise comparison

Figure 2 presents that the area under the curve (AUC) was 0.503, indicating that the model performs almost no better than chance — in other words, FullPiers did not discriminate well between patients with and without eclampsia in this sample. The optimal cutoff point for the FullPiers score was approximately 0.044, with: Sensitivity: 0.38 (37.5%) Specificity: 0.91 (90.8%). FullPiers values ≥0.044 identify about 38% of eclampsia cases, while 91% of non-eclampsia cases are correctly classified as negative.

**Figure 2:**
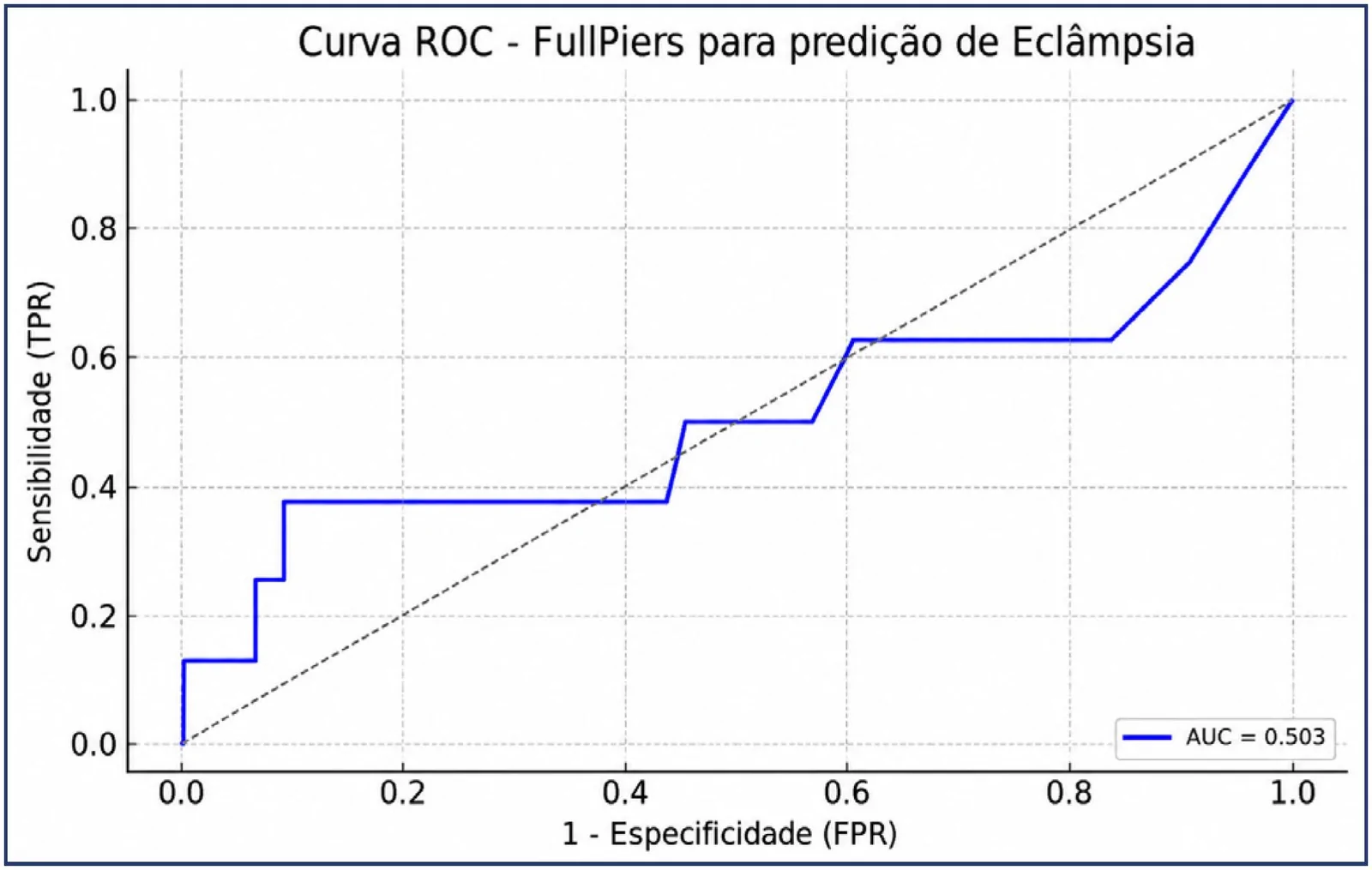
AUC – FullPiers for Eclampsia Prediction

## Discussion

Our findings support the impact of medically indicated preterm birth due to preeclampsia in a referral center, accounting for around 10% of all deliveries in the study period. Among these cases, there was increased maternal and perinatal morbidity, presenting: higher frequency of thrombocytopenia, elevated liver enzymes, and increased markers of renal dysfunction, findings that suggest greater severity of hypertensive disease and a high systemic involvement. Regarding obstetric outcomes, higher frequency of newborns with very low birth weight and a higher occurrence of fetal death.

Describing maternal and perinatal outcomes due to preterm PE is relevant, since scientiﬁc evidence indicates that the underlying mechanism of PE, characterized by endothelial dysfunction, is not limited to pregnancy. This condition may reduce life expectancy in affected women, increasing the risk of stroke, cardiovascular disease, and diabetes up to sixfold.^(8,9)^ Moreover, infants born from pregnancies complicated by PE are at higher risk of preterm birth, perinatal death, neurodevelopmental impairments, and long-term susceptibility to cardiovascular and metabolic diseases, especially in early onset disease (prior to 34 weeks of gestation).^(3,4,8)^

Delivery of the feto-placental unit and deciding on the timing of childbirth represents one of the greatest challenges in obstetric practice, especially in cases of early-onset PE. In this context, the fullPIERS calculator, an available decision-support tool, is designed to identify pregnant women at increased risk of adverse outcomes and to predict complications occurring within 48 hours to 7 days after assessment.^(10)^ It considers factors such as gestational age, chest pain, dyspnea, oxygen saturation, platelet count, creatinine, and liver enzymes.^(9,11)^

When considering the use of the fullPIERS, which had an average of 2.30, about one quarter of the women exceeded the cutoff point of 2.15. This cut-off has been described in previous studies in Brazil, however not yet implemented in clinical practice. The established cutoff is relevant, as it correlates with adverse laboratory markers such as thrombocytopenia and elevated liver transaminases, and also with worse obstetric outcomes, confirming the validity of the score in risk stratification of patients with PE.^(10,11)^ Validating its use is important to provide better training and follow-up, especially for early onset PE, which is associated to worse outcomes.

The indications for preterm birth in our study showed significant variations, with refractory blood pressure peak being the main cause, almost three-quarters of the cases analyzed, which is consistent with the literature.^(1,2,7)^ Administration of magnesium sulfate was observed in 87% of cases—84.9% in the group with fullPIERS <2.15% and 96.3% in the group ≥2.15%. However, it was not possible to further distinguish in which patients magnesium sulfate was used and in which it was used for eclampsia prevention.^(3,8)^ In addition, an average of 4.18 doses of hydralazine were administered before pregnancy termination. Notably, laboratory conditions such as HELLP syndrome and abnormal test results showed a strong association with scores above 2.15 (p<0.001), emphasizing that beyond hypertension control, an integrated approach that considers laboratory parameters when deciding the appropriate timing for pregnancy termination is crucial.^(4,9)^

As expected, length of hospital stay was related to the severity of maternal condition, though the analysis revealed variable trends. The group of women who delivered "very preterm" (28–31+6 weeks) had the shortest length of stay (6.0 days), suggesting faster disease progression requiring earlier delivery. In contrast, the "moderate preterm" group (32–33+6 weeks) had the longest hospital stay (11.4 days), possibly reflecting early/severe preeclampsia with good inpatient management, resulting in prolonged hospitalization despite eventual preterm delivery. The "late preterm" group (34–36+6 weeks) showed an intermediate length of stay (7.13 days), with an overall mean hospitalization time of 7.7 days.

The recently updated protocol of the Brazilian network for studies on hypertension in pregnancy (RBEHG)^(7)^ suggests hospitalization for the management of diagnosed PE cases, especially in severe features, requiring magnesium sulfate treatment. However, regional disparities in low- and middle-income countries like Brazil must be considered, regarding infrastructure, bed availability, healthcare teams, and access to high-risk referral services, understanding that in many public services it is not possible to maintain hospitalization for all patients. In the considered referral maternity, the implemented protocol advice for admission of all PE cases, especially if preterm/early onset, allowing for early and timely intervention in severe cases, demonstrated by the low incidence maternal mortality rates related to hypertensive syndromes, with no cases reported in the last 12 years in this high-risk service. Thus, demonstrating that the possibility of hospitalization for all patients with PE, combined with risk calculations, like FullPIERS, may be a great ally in reducing maternal mortality.

In a previous review, about five years ago, using studies performed in Brazil, considering 52.986 pregnant women, the prevalence of PE was 6.7%, resulting in 2.988 reported cases.^(12-19)^ A lower prevalence than that found in this study, which showed a PE rate of 9.75% In this study, among preterm PE cases, the mean gestational age at delivery was approximately 34 weeks, with the group fullPIERS ≥2.15 averaging 32.8 weeks and the group <2.15 averaging 34.4 weeks. There was a higher frequency of moderate prematurity and a greater proportion of neonates weighing <1500 g at birth. Regarding neonatal outcomes, 15.7% were born weighing <1500 g and the stillbirth rate was 2.45%. All stillbirths occurred before 31 weeks of gestation (>50% at <28 weeks). These results are in accordance to the literature, with worse outcomes among cases of Early onset PE.^(6,12,13)^

The idea to test the accuracy of the fullPIERS model to predict eclampsia was suggested considering that this complication is still the most significant burden in terms of mortality due to PE in Brazil. Nevertheless, the fullPIERS model alone showed limited accuracy for predicting the condition in this study, with an AUC of 0.503 close to random chance. At the optimal Youden index cutoff (0.044), specificity was high (90.8%), but sensitivity was low (37.5%). One of the reasons for the poor performance of the ROC curve is that the only available outcome used was eclampsia. This means the model reliably ruled out eclampsia, reducing unnecessary interventions in resource-limited settings. External validations show similar trends, with specificity between 80–90% and variable sensitivity, highlighting the need for local recalibration before broader use.^(17-19)^ Overall, FullPIERS may be valuable as a supplementary tool for ruling out risk in pre-eclampsia patients. Further studies should add other relevant adverse outcomes such as stroke, hypertensive encephalopathy, coma, disseminated intravascular coagulation (DIC), need for ICU and maternal death.

The rate of preterm birth associated with preeclampsia represents a major challenge in obstetrics, being responsible for key neonatal complications, including respiratory distress, intraventricular hemorrhage, and necrotizing enterocolitis, as well as potential long-term effects on infant development. It is estimated that worldwide, 300 million women and children face chronic health risks due to prior exposure to preeclampsia.^(4)^ In Brazil, according to the World Health Organization (WHO), the preterm birth rate is approximately 12%, with 37% of these births resulting from obstetric intervention.^(12,13)^ Globally, about 15% of preterm births are attributable to preeclampsia. In Brazil, the disease accounts for roughly 18% of all preterm deliveries, whereas in developed countries, preterm births associated with preeclampsia represent about 8% of total preterm births.^(14)^

One of the strengths of this study is that, since the fullPIERS calculation was performed after the pregnancy outcome, fetal and neonatal variables, which are not usually assessed in risk calculation, were evaluated. Additionally, through a reverse approach, first resolving the pregnancy and then correlating it with the risk calculation, the practical applicability of the calculator was also demonstrated.

However, some limitations of this study must be acknowledged, such as its retrospective nature, which limits the control and standardization of data collection. Being a single-center study with a small N value, it limits generalization to larger populations. Furthermore, the risk calculation assessment was carried out in a reference center that does not routinely use it in practice, which posed limitations in collecting information that would usually be necessary for risk calculation.

Overall, these findings highlight and validate the fullPIERS score cutoff ≥ 2.15 as a valuable tool for risk stratification, enabling the identification of women with greater clinical severity and a higher likelihood of adverse outcomes. This study contributes to the literature by demonstrating the applicability of fullPIERS in women who underwent medical interventions resulting in preterm delivery in the Brazilian context, providing a foundation for more precise clinical decision-making and for integrating laboratory indicators into clinical healthcare. The overall data on maternal and perinatal outcomes in medically indicated preterm birth due to PE support health policy interventions, in order to increase training, accurate diagnosis and referral.

## Conclusion

Preeclampsia-related prematurity remains a major contributor to adverse perinatal outcomes and is a frequent indication for medically indicated preterm delivery. Decisions regarding early pregnancy interruption are often driven by maternal clinical or laboratory deterioration, in this context, risk prediction tools such as the fullPIERS calculator may represent an important tool when used to support comprehensive clinical management. It is important to emphasize that the model does not incorporate perinatal outcomes or warning maternal clinical signs, including symptoms suggestive of imminent eclampsia, which may limit its ability to fully capture disease severity. Therefore, particularly in preterm gestations, risk estimates should be interpreted cautiously and integrated with careful clinical assessment, reinforcing that predictive calculators should complement – rather than replace – individualized clinical judgment when balancing maternal safety against the consequences of prematurity.

## Data Availability

The research data are described in the article presented.
